# Engaging youth as citizen scientists to determine health needs of New Brunswick adults

**DOI:** 10.1017/cts.2023.674

**Published:** 2023-11-15

**Authors:** Sara W. Heinert, Joanne Ciezak, Jeremiah Clifford, Tamara Cunningham, Affan Aamir, Ananya Penugonda, Shawna V. Hudson

**Affiliations:** 1 Department of Emergency Medicine, Rutgers Robert Wood Johnson Medical School, Rutgers University, New Brunswick, NJ, USA; 2 New Brunswick Health Sciences Technology High School, New Brunswick, NJ, USA; 3 System Development/Planning, RWJBarnabas Health, Somerset, NJ, USA; 4 Department of Family Medicine and Community Health, Rutgers Robert Wood Johnson Medical School, Rutgers University, New Brunswick, NJ, USA

**Keywords:** Adolescents, community health needs assessment, public health, health promotion, community health

## Abstract

Community health needs assessments (CHNAs) are important tools to determine community health needs, however, populations that face inequities may not be represented in existing data. The use of mixed methods becomes essential to ensure the needs of underrepresented populations are included in the assessment. We created an in-school public health course where students acted as citizen scientists to determine health needs in New Brunswick, New Jersey adults. By engaging members of their own community, students reached more representative respondents and health needs of the local community than a CHNA completed by the academic hospital located in the same community as the school which relies on many key health statistics provided at a county level. New Brunswick adults reported significantly more discrimination, fewer healthy behaviors, more food insecurity, and more barriers to accessing healthcare than county-level participants. New Brunswick participants had significantly lower rates of health conditions but also had significantly lower rates of health screenings and higher rates of barriers to care. Hospitals should consider partnering with local schools to engage students to reach populations that face inequities, such as individuals who do not speak English, to obtain more representative CHNA data.

## Introduction

A community health needs assessment (CHNA) examines a given population’s health status to identify a community’s key problems and assets, and create strategies to address health needs and identified issues [1]. As part of The Patient Protection and Affordable Care Act of 2010, nonprofit hospitals must complete a CHNA every three years to claim their tax-exempt status [2]. In 2022, a large academic hospital in New Brunswick, New Jersey (NJ)- Robert Wood Johnson University Hospital (RWJUH), in partnership with its neighboring hospital, Saint Peter’s University Hospital, and Healthier Middlesex (a diverse, multi-sector, community-focused consortium) conducted a CHNA of the communities it serves in Middlesex County, NJ [3]. The report [3] provides rich data on the hospital’s primary service area; however, given NJ’s socioeconomic, racial/ethnic, and rural/urban diversity, there are likely local health needs important for planning local interventions that may not be well-represented with the reliance on larger county-level health statistics. Additionally, populations that face greater inequities, such as individuals who do not speak English, may not be represented in existing community data [4].

A citizen science model can engage directly with individual community members to collect data, interpret findings, and implement dissemination/advocacy [5]. A review of 27 articles describing citizen science projects found that the most common areas of application were environmental contaminant exposures, physical activity, and healthy eating [6]. Authors of the review recommended expanding the focus on topics important for health equity [6]. Citizen science can be especially relevant for fostering health equity as health inequities are often best understood by those experiencing them and lesser known by those who control a community’s decision-making channels [5]. Engaging youth in health assessments has shown increased youth empowerment and leadership potential [7]. Of greatest importance is increasing youth engagement in health and health equity in communities of color.[7] Youth who contribute to health programs and services can also expand their own knowledge and increase their healthful decision-making capacity [8]. Additionally, health inequities can be addressed by increasing diversity in the primary care workforce to better reflect the experiences of the communities served [9]. Thus, hands-on health opportunities can increase youths’ interest in pursuing health careers with the potential to contribute to a more diverse future healthcare workforce. Activating the underutilized resource of engaging youth as active participants and potential drivers of positive change in the community could be beneficial for participants and their communities with the opportunity to advance health equity [10].

Partners from an academic medical school and a community high school worked together to develop an innovative hands-on public health course at a local high school. This manuscript describes the creation of the course where high school students act as citizen scientists to engage community members to obtain information on health needs and barriers for New Brunswick adults in an effort to address health equity. We also wanted to determine if students were able to reach participants who were more representative of the local community than characteristics of participants in the larger hospital assessment.

## Methods

New Brunswick Health Sciences Technology High School (NBHSTHS) is a specialized high school located steps from Robert Wood Johnson Medical School and Robert Wood Johnson University Hospital in New Brunswick, New Jersey. New Brunswick is an urban setting where over half of residents are Hispanic [11] and about one-third live in poverty [12]- The student population is approximately 200 students and is predominately Hispanic. The school is designed to prepare youth for the challenges of a career in medicine and health care and students have the opportunity to shadow healthcare workers at RWJUH, as well as work at the hospital over the summer.

Development of the high school public health course was the result of a new community-academic collaboration between Rutgers Robert Wood Johnson Medical School (RWJMS) and New Brunswick Health Sciences Technology High School (NBHSTHS) that began in late 2021. The RWJMS faculty member worked with the school’s Principal and Director of Curriculum and Instruction to co-create the course, as there was previously no course or curriculum about public health at the school. The course was especially relevant given the COVID-19 pandemic. At the start of the course, the school and hospital resumed their partnership with students participating in clinical learning opportunities in the hospital. However, many students and their parents did not yet feel comfortable going to hospital during the pandemic, especially given the Omicron surge at the time. The course provided a hands-on public health learning opportunity for these students in a non-clinical setting. At the same time, collection of community health needs data was especially timely given the potential shift in health utilization and needs, as well as healthy behaviors, during the pandemic.

In Spring 2022 (January–June 2022), 26 Juniors at New Brunswick Health Sciences Technology High School participated in a for-credit, in-school public health class with experiential learning that met 2.5 hours/week for 16 weeks. Curriculum included public health topics and careers, health disparities, basic statistics, and socioeconomic data on New Brunswick. Specific topics for each class are found in Table 1. The course was predominately taught by the co-leaders of the project (both academic and community leaders) with some teaching by undergraduate and medical students from the academic partner. For a hands-on assignment, each student completed 10 CHNAs with adults whom they knew (family or friends) in their community (New Brunswick, NJ) using the 30-question 2022 RWJUH CHNA, available in both English and Spanish. Assessment data were collected by students and entered into and managed via the secure, web-based software platform REDCap (Research Electronic Data Capture) hosted at Rutgers University [13]. Students collected no names or contact information with the data and used a unique code to get class credit for each survey collected, which was then deleted from the dataset prior to analysis. To compare student CHNA data in New Brunswick to hospital CHNA data in Middlesex County, NJ, we pulled hospital CHNA data from the publicly available 2022 CHNA Report [3]. Data were analyzed using proportion tests with Stata version 16.0 (StataCorp). Additionally, at the end of the course. students were asked to provide anonymous course feedback to improve learning for future cohorts. This project was determined to be non-human subject research by the Rutgers University Institutional Review Board, as it was an assignment as part of the public health class at school. The curriculum was submitted and approved by the District Curriculum Committee.

**Table 1.** Spring 2022 class curriculum

Week	Topic
1	Intro to Public Health
2	Epidemiology
3	Journal Articles
4	Risk Factors
5	Prevention
6	Access, Quality, and Cost/Healthcare Access
7	Asset Mapping/Assets in New Brunswick
8	Survey Design/Community Health Assessments
9	Health Disparities- Part 1
10	Health Disparities- Part 2/Disparities in New Brunswick
11	Numbers in Public Health- Variables, Associations, Graphs, Basic Statistics
12	Community Health Assessment Data Review, Asking Questions, Conducting Interviews
13	Work on Group Presentations in Class
14	Work on Group Presentations in Class
15	Group Presentations to Class
16	Group Presentations to Class

## Results

Students successfully completed 201 CHNAs with New Brunswick adults, of which 21% (43) were completed in Spanish. Table 2 shows demographic characteristics of New Brunswick and Middlesex County residents based on Census data, demographics of CHNA participants completed by students and by the hospital, as well as statistically significant differences between findings in both CHNAs. New Brunswick residents and CHNA participants were significantly younger, more often single, had less education, and had lower household income, with more Hispanic and less White and Asian residents/participants than Middlesex County residents and hospital CHNA participants.

**Table 2.** Demographic characteristics of new brunswick and middlesex county and comparison of student (N = 201) and hospital (N = 556) community health needs assessment participant characteristics

Participant Characteristics		% in New Brunswick^^^	% in Middle- sex County^^^	Student CHNA-New Brunswick %, 95% CI	Hospital CHNA- Middlesex County %	p-value*
Population/Sample Size		55,671	858,770	201	556	
Age (years)	18–29	37.0	15.6	35.3 (28.7–41.9)	11.3	**<0.001**
	30–49	23.9	27.4	47.3 (40.4–51.2)	32.0	**<0.001**
	50–64	10.6	20.1	11.5 (7.0–15.8)	36.4	**<0.001**
	65+	6.4	15.1	3.5 (0.9–6.0)	20.4	**<0.001**
	Prefer not to answer	−	−	2.5	−	−
Gender identity	Male	50.5	49.6	38.5 (31.8–45.2)	30.9	**0.02**
	Female	49.5	50.4	55.5 (48.6–62.4)	68.4	**<0.001**
	Transgender/Other	NA	NA	1.5 (−0.2–3.2)	0.7	0.18
	Prefer not to answer	−	−	5.0	−	−
Ethnic/ racial background (check all that apply)	African American	11.4	9.1	7.5 (3.8–11.1)	9.1	0.42
	Asian	10.4	26.4	1.5 (−0.2–3.2)	10.9	**<0.001**
	Caucasian/White	18.7	38.6	5.0 (2.0–8.0)	62.4	**<0.001**
	Hispanic, Latino(a)	56.8	22.4	81.6 (76.2–86.9)	12.2	**<0.001**
	Other	1.5	1.0	2.5 (0.3–4.6)	5.4	0.07
	Prefer not to answer	−	−	6.0		
Marital status	Single	66.2	33.0	45.3 (38.4–52.2)	22.5	**<0.001**
	Married	23.2	52.8	40.3 (33.5–47.1)	60.4	**<0.001**
	Separated/ divorced/ widowed	10.6	14.1	6.0 (2.7–9.2)	12.2	**0.01**
	Domestic partnership/civil union/living together	NA	NA	8.5 (4.6–12.3)	4.9	**0.02**
Highest level of education (from Census- population 25+ yrs)	Less than high school graduate or GED	34.1	10.1	33.0 (26.5–39.5)	1.3	**<0.001**
	High school graduate or GED	26.7	24.0	25.5 (19.5–31.5)	9.7	**<0.001**
	Some college	10.8	14.6	16.0 (10.9–21.1)	13.7	0.34
	Associate or technical degree/certification	3.8	6.5	7.0 (3.5–10.5)	11.7	**0.04**
	College graduate	13.6	26.5	9.5 (5.4–13.6)	32.1	**<0.001**
	Post-graduate or professional degree	11.0	18.3	0.5 (−0.5–1.5)	31.7	**<0.001**
	Prefer not to answer	−	−	9.0	−	−
Current employment status (Census- unemployment rate)	Employed full-time	NA		62.2 (55.5–68.9)	60.9	0.61
	Employed part-time			15.4 (10.4–20.4)	9.1	0.09
	Student			10.9 (6.6–15.3)	4.3	**<0.001**
	Homemaker			5.5 (2.3–8.6)	3.3	0.09
	Disabled			1.5 (-0.2–3.2)	3.4	0.14
	Retired			2.5 (0.3–4.6)	13.9	**<0.001**
	Unemployed	6.5	6.1	4.0 (1.3–6.7)	5.0	0.51
	Prefer not to answer			7.0		
Household income	Under $25,000	30.5	11.1	10.6 (6.3–14.8)	5.5	**0.002**
	$25,000–$50,000	19.9	13.6	32.2 (25.7–38.7)	13.6	**<0.001**
	$50,001–$100,000	25.4	26.7	27.1 (21.0–33.3)	33.0	0.08
	$100,001–$150,000	12.1	20.0	3.5 (1.0–6.1)	24.2	**<0.001**
	$150,001–$200,000	5.9	12.5	0.5 (−0.5–1.5)	9.2	**<0.001**
	Over $200,000	6.2	16.0	0.5 (−0.5–1.5)	14.5	**<0.001**
	Prefer not to answer	−	−	26.4	−	

NA = Not Available.

^ All data from U.S. Census Bureau, 2017–2021 American Community Survey 5-Year Estimates except race/ ethnicity data from U.S. Census Bureau, 2020 Census Redistricting Data (Public Law 94–171).

* Student vs. hospital CHNA.

Table 3 compares findings of the two CHNA respondent groups. Adults in the student CHNA reported significantly more discrimination compared to the hospital CHNA for language or speech (*p* < .001), race or ethnicity (*p* < .001), cultural or religious background (*p* < .001), and income level (*p* < .001). Compared to the hospital CHNA, student CHNA participants reported significantly fewer healthy behaviors (healthy eating and physical activity) (*p* < 0.001 for all questions), and more food insecurity (*p* < 0.001 for all questions). Student CHNA participants had significantly lower rates of health conditions for all conditions except asthma but also had significantly lower rates of health screenings (*p* < 0.001 for all screenings) and higher rates of barriers to care (with the most prevalent being insurance problems (46.8%, *p* < .001) and cost of care (44.3%, *p* < .001)), suggesting participants may be unaware of undiagnosed health conditions.

**Table 3.** Comparison of student (N = 201) and hospital (N = 556) community health needs assessment data

		Student CHNA %, 95% CI	Hospital CHNA, %	p–value
**Community assets** (% Agree or Agree Completely)	My community has places for everyone to socialize.	54.3 (47.4–61.3)	45.7	**0.02**
	It’s easy to find fresh fruits and vegetables in my community.	46.4 (39.4–53.4)	55.0	**0.02**
	There are job opportunities in my area.	43.4 (36.4–50.3)	46.2	0.43
	My community has safe outdoor places to walk and play.	37.8 (31.0–44.7)	82.2	**<0.001**
	It’s easy to live a healthy lifestyle in my community.	33.7 (27.1–40.3)	38.7	0.15
	My community is a good place to raise a family.	32.7 (26.1–39.2)	49.6	**<0.001**
	My community has transportation services that assist residents in getting to doctor appointments.	30.3 (23.8–36.7)	32.0	0.60
	There are educational opportunities for adults in my community.	24.9 (18.8–30.9)	48.6	**<0.001**
	People in my community can afford basic needs like food, housing, and transportation.	22.8 (17.0–28.7)	51.4	**<0.001**
	There is enough affordable housing that is safe and well–kept in my community.	20.6 (14.9–26.3)	37.0	**<0.001**
	Violence is not prevalent in my community.	19.0 (13.5–24.5)	37.8	**<0.001**
**Discrimination** ^1^ (% Yes)	Language or speech	39.6 (32.8–46.4)	7.7	**<0.001**
	Race or ethnicity	32.0 (25.5–38.5)	12.3	**<0.001**
	Cultural or religious background	16.1 (11.0–21.2)	6.9	**<0.001**
	Income level	16.1 (11.0–21.2)	9.0	**<0.001**
**Health screenings received (past 2 years)** (% Yes)	Cholesterol screening	42.9 (36.0–49.8)	70.9	**<0.001**
	Blood pressure check	57.1 (50.2–64.1)	87.9	**<0.001**
	Flu shot	62.6 (55.8–69.4)	75.2	**<0.001**
	Dental screening (e.g., x–rays, cleaning, etc.)	62.4 (55.7–69.2)	76.3	**<0.001**
	Annual physical exam	57.9 (51.0–64.8)	81.3	**<0.001**
**Healthy behaviors** (% Yes)	Do you feel that you eat healthy foods on a regular basis?	46.7 (39.8–53.7)	82.4	**<0.001**
	Do you feel that you have enough information to understand what food is healthy?	55.3 (48.4–62.2)	93.9	**<0.001**
	Do you feel that you are physically active?	41.5 (34.7–48.3)	70.3	**<0.001**
**Food security (last 12 MOS)** (% Sometimes or Often True)	We worried whether our food would run out before we got money to buy more.	55.3 (48.4–62.3)	20.3	**<0.001**
	The food that we bought just didn’t last and we didn’t have money to get more.	43.9 (37.0–50.9)	14.7	**<0.001**
	We rely on a community supper program, food pantry or meal assistance program to supplement our household.	37.9 (31.1–44.6)	11.3	**<0.001**
**COVID-19** (% Yes)	Self or anyone in immediate family lost employment due to COVID-19	31.8 (25.4–38.3)	27.4	0.16
	*Since COVID-19 started, have you or anyone in your immediate family personally experienced any difficulty with:*			
	Maintaining good physical health	58.2 (51.4–65.0)	43.8	**<0.001**
	Maintaining good mental state	53.7 (46.8–60.6)	43.8	**0.01**
	Feeling lonely or isolated from others	24.9 (18.9–30.9)	35.4	**0.002**
**Health conditions** ^2^ (% Yes)	High blood pressure	32.8 (26.3–39.4)	61.0	**<0.001**
	High cholesterol	28.3 (22.0–34.6)	51.0	**<0.001**
	A weight problem	28.6 (22.3–34.9)	55.0	**<0.001**
	Asthma	26.9 (20.7–33.1)	27.2	0.93
	Diabetes	26.6 (20.5–32.8)	34.5	**0.02**
	Depression or anxiety issues	22.1 (16.3–27.9)	42.6	**<0.001**
**Community perceptions of health issues/ concerns** ^3^	Overweight/obesity	45.8 (38.9–56.7)	36.4	**0.01**
	Many cases of diabetes	30.3 (24.0–36.7)	11.4	**<0.001**
	Mental health issues	28.9 (22.6–35.1)	30.3	0.66
	Substance use, abuse, or overdose	25.4 (19.4–31.4)	14.4	**<0.001**
	Smoking/vaping	23.9 (18.0–29.8)	11.5	**<0.001**
	High stress lifestyle	15.4 (10.4–20.4)	21.3	0.04
**Barriers to accessing healthcare services** ^4^	Insurance problems	46.8 (39.9–53.7)	23.5	**<0.001**
	Cost of care	44.3 (37.4–51.1)	22.0	**<0.001**
	Ability to schedule an appointment at a convenient time	36.3 (29.7–43.0)	28.0	**0.01**
	Wait times at doctor’s office or clinic are too long	27.9 (21.7–34.1)	21.3	**0.02**
	Never experienced any difficulty in getting care	13.4 (8.7–18.1)	35.8	**<0.001**

1 When trying to receive medical care, you or a family member Personally felt discriminated against, based on the following characteristics.

2 Have you, or a household family member, ever been told by a doctor or other health professional that you have had any of the following?.

3 In your opinion, what are the top 3 health issues or concerns in your community?.

4 Over the last few years, which, if any, of these issues made it difficult for you or a household family insurance or you do not have any insurance) member, to get medical treatment or care when needed?

### Student Feedback

At the end of the Spring 2022 class, students were asked to answer four questions in REDCap in order to improve future renditions of the class: (1) What did you like most about the class?, (2) What did you like least about the class?, (3) What would you change about the class?, and (4) Is there anything you didn’t learn in the class but wanted to learn?. Twenty-two (22) of the 26 students completed the feedback assessment. Overall, students enjoyed the content of the class and learned a lot. They especially enjoyed learning about the health of their community, determining solutions to problems, and doing so in a unique way that explored them in-depth. Some specific feedback from students is below:

*“What I liked most about this class is that we were able to learn about public health/ new topics that we never talked about/ or never cared about.”*

*“I liked the most that this class taught the problems in the community and let us brainstorm future solutions as future leaders.”*

*“I really enjoyed how interactive the class was, whether it be interviews, collecting irl [in real life] data, or making presentations with the collected data.”*

*“I would say I enjoyed making the slide presentation about a problem in the health sector in New Brunswick. I was able to study the data and come up with some type of solution.”*

*“I like the information provided in the class. Like what one would do if they were to be in Public health and the different problems faced by Public Health Workers.”*

The most common criticism from students was that the class was too long at 3 class periods, which was cited by 55% of students, although two students said they wanted the class to meet more often- one wanted it to meet more than once per week and one wanted it to meet the full year rather than half the year. Four students (18%) suggested making the class more engaging or interactive with more class participation.

Ten students (45%) said there was nothing they wanted to learn in the class that they did not learn. Four students (18%) wanted to learn more about public health careers/pathways and three (14%) wanted to learn more about additional communities than only New Brunswick.

## Discussion

By engaging members of their own community, students reached more representative respondents and health needs of the local community than a county-level CHNA completed by the academic hospital where the school is located.

CHNAs aim to use the data to plan, implement, and evaluate strategies to create a healthier community. In class, with guidance and oversight from project leadership, students reviewed CHNA data and brainstormed 14 questions to ask New Brunswick adults to obtain more in-depth information on health barriers and needs, such as “How has COVID-19 affected your daily life?.” As a homework assignment, students interviewed two New Brunswick adults with 5 questions of their choosing, for a total of 52 interviews completed. Based on the surveys and interviews, students chose 6 health issues that were most relevant to New Brunswick. These issues were: mental health, costs of healthcare, obesity, diabetes, physical health & COVID-19, and language barriers in healthcare. Students were put into groups and presented on a New Brunswick health issue including an explanation of the problem and an intervention to address the problem. The second cohort of the course occurred in Fall 2022 with 16 students. These students followed the public health curriculum but focused on diabetes- as it was determined to be a major health issue in the community. For their hands-on project, the students organized and ran a health fair at the school, which had 36 participants. We will continue to engage youth to determine how they can support improving the health of their community.

Additionally, the content of the class is constantly evolving. For example, in the past, we have focused on students learning about public health topics and learning about health issues in their community, without much discussion on research methodology. Future renditions of the course can include more emphasis on training in research methods and ethics.

Collection of community health needs data is especially timely given the potential shift in health utilization and needs, as well as healthy behaviors, during the pandemic, and involving high school students as collectors of the data is innovative. Academic and community partners mutually benefit from student engagement as citizen scientists in providing a more robust representation of local health needs which can be effectively targeted for future interventions. Students also benefit from their engagement to help determine the health needs of New Brunswick, thus increasing their health literacy, understanding of health infrastructure, and empowering their advocacy for health program improvement. This experience has increased interest in continuation and expansion of this model as both an educational tool for students and as a valuable source of input to the hospital for understanding local health needs.

This project successfully provided a surveillance of local health needs and barriers to inform future health interventions for New Brunswick while engaging youth to drive their own inquisition about their community’s health. Hospitals should consider partnering with local schools to engage students to reach populations that face inequities, such as individuals who do not speak English, to obtain more representative CHNA data. Findings from this pilot project can be used to expand the model to additional schools in other communities where local health needs and barriers may not be represented in existing community data.
